# A Methylazanediyl Bisacetamide Derivative Sensitizes *Staphylococcus aureus* Persisters to a Combination of Gentamicin And Daptomycin

**DOI:** 10.1002/advs.202306112

**Published:** 2023-12-21

**Authors:** Hee Young Heo, Guijin Zou, Seongeun Baek, Jae‐Seok Kim, Eleftherios Mylonakis, Frederick M. Ausubel, Huajian Gao, Wooseong Kim

**Affiliations:** ^1^ College of Pharmacy Graduate School of Pharmaceutical Sciences Ewha Womans University Seoul 03760 Republic of Korea; ^2^ Institute of High Performance Computing (IHPC) Agency for Science Technology and Research (A*STAR) Singapore 138632 Republic of Singapore; ^3^ Department of Laboratory Medicine Kangdong Sacred Heart Hospital Hallym University College of Medicine Seoul 05355 Republic of Korea; ^4^ Department of Medicine Houston Methodist Hospital Houston TX 77030 USA; ^5^ Department of Molecular Biology Massachusetts General Hospital Boston MA 02114 USA; ^6^ Department of Genetics Harvard Medical School Boston MA 02115 USA; ^7^ School of Mechanical and Aerospace Engineering College of Engineering Nanyang Technological University Singapore 639789 Republic of Singapore

**Keywords:** antibiotic resistance, antibiotic tolerance, Caenorhabditis elegans, membrane‐active antimicrobials, methylazanediyl bisacetamide, MRSA, persisters, synergism

## Abstract

Infections caused by *Staphylococcus aureus*, notably methicillin‐resistant *S. aureus* (MRSA), pose treatment challenges due to its ability to tolerate antibiotics and develop antibiotic resistance. The former, a mechanism independent of genetic changes, allows bacteria to withstand antibiotics by altering metabolic processes. Here, a potent methylazanediyl bisacetamide derivative, MB6, is described, which selectively targets MRSA membranes over mammalian membranes without observable resistance development. Although MB6 is effective against growing MRSA cells, its antimicrobial activity against MRSA persisters is limited. Nevertheless, MB6 significantly potentiates the bactericidal activity of gentamicin against MRSA persisters by facilitating gentamicin uptake. In addition, MB6 in combination with daptomycin exhibits enhanced anti‐persister activity through mutual reinforcement of their membrane‐disrupting activities. Crucially, the “triple” combination of MB6, gentamicin, and daptomycin exhibits a marked enhancement in the killing of MRSA persisters compared to individual components or any double combinations. These findings underscore the potential of MB6 to function as a potent and selective membrane‐active antimicrobial adjuvant to enhance the efficacy of existing antibiotics against persister cells. The molecular mechanisms of MB6 elucidated in this study provide valuable insights for designing anti‐persister adjuvants and for developing new antimicrobial combination strategies to overcome the current limitations of antibiotic treatments.

## Graphical abstract

This study presents MB6, a methylazanediyl bisacetamide derivative, as a potent antimicrobial agent selectively targeting MRSA membranes. MB6 demonstrates triple synergistic killing against MRSA persisters by promoting gentamicin uptake and enhancing the membrane‐disrupting activity of daptomycin. This positions MB6 as a promising adjuvant in combating antibiotic‐resistant and ‐tolerant infections, offering innovative strategies in antimicrobial treatments.

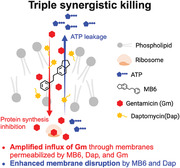

## 1 Introduction

The ability of bacteria to survive high concentrations of antibiotics without genetic alterations is known as antibiotic tolerance, a phenomenon that differs from antibiotic resistance.^[^ ^1^ ^]^ Unlike resistance, which is genetically acquired and enables bacteria to proliferate under antibiotic pressure, tolerance is a non‐heritable transient characteristic. This trait allows bacteria to survive lethal antibiotic concentrations by a variety of mechanisms including the reduction of metabolic activity or entering a non‐growing dormant state.^[^ ^2^ ^]^

Bacteria demonstrating antibiotic tolerance, known as persisters, can endure various types of antibiotics with distinct mechanisms of action. Persisters are often found in stationary‐phase cell populations or biofilms where they are protected by a self‐secreted substance matrix.^[^ ^2^, ^3^ ^]^ In clinical settings, persisters contribute to prolonged infection courses, treatment failures, and antibiotic resistance development, as seen in conditions such as recurrent urinary tract infections, chronic infections in cystic fibrosis patients, and endocarditis.^[^ ^1b,c^ ^]^ These challenges highlight the need for new therapeutic strategies.

Persisters exhibit tolerance to different types of bactericidal inhibitors targeting the bacterial cell wall, nucleic acids, or protein synthesis. In the case of inhibitors of cell wall or nucleic acid biosynthesis, antibiotic tolerance typically arises due to a significant reduction in the rate of biosynthesis of the cell wall or nucleic acids.^[^ ^4^ ^]^ In contrast, for aminoglycosides, a bactericidal class of protein synthesis inhibitors, tolerance is typically achieved by impeding antibiotic uptake due to diminished proton motive force (PMF), which is essential for the cellular entry of aminoglycosides.^[^ ^5^ ^]^ Interestingly, because proteins are still synthesized in bacterial persisters, albeit at decreased levels,^[^ ^5^, ^6^ ^]^ the use of adjuvants that promote aminoglycoside uptake can successfully eradicate these cells. For instance, carbohydrate metabolites and adenosine have been shown to restore the bactericidal activity of aminoglycosides by increasing PMF in persister cells of *Staphylococcus aureus*, a major human pathogen known to readily acquire antibiotic resistance and form antibiotic‐tolerant persisters.^[^ ^5^, ^7^ ^]^ Moreover, membrane‐disrupting antimicrobial agents including the synthetic retinoid CD437, the anthelmintic bithionol, or biosurfactant rhamnolipids have been demonstrated to enhance the potency of aminoglycosides against antibiotic‐resistant *S. aureus* persisters by promoting PMF‐independent uptake.^[^ ^4^, ^8^ ^]^

Daptomycin, a lipopeptide antibiotic, forms pores in membranes with the assistance of calcium ions,^[^ ^9^ ^]^ which ultimately leads to membrane perturbation and subsequent bacterial death. As the bacterial membrane is crucial for viability, regardless of metabolic or growth states, daptomycin can be partially effective against *S. aureus* persisters. However, a subpopulation of bacteria may still exhibit tolerance to daptomycin and remain viable.^[^ ^8^, ^10^ ^]^ Moreover, the bactericidal potency of daptomycin is significantly decreased against persisters formed by certain antibiotic‐resistant *S. aureus* strains.^[^ ^8^, ^11^ ^]^ Despite these limitations, the activity of daptomycin can be enhanced when paired with adjuvants like d‐cycloserine. This compound modifies the membrane change, which in turn augments the affinity between daptomycin and bacterial membranes.^[^ ^12^ ^]^ Insights from these membrane‐targeting compounds propose they could potentially serve not only as standalone therapeutics, but also as adjuvants for traditional antibiotics.

In this study, we report on a novel methylazanediyl bisacetamide derivative, MB6, that exhibits membrane‐active antimicrobial properties with a selectivity toward bacterial over mammalian membranes. MB6 sensitizes MRSA persisters to both gentamicin and daptomycin. Notably, the triple combination of these three agents exerts an enhanced lethal effect on MRSA persisters, far outperforming each of the individual agents alone or each of the two‐agent combinations. Our investigation highlights the potential of selective membrane‐active agents such as MB6 to serve as effective antimicrobials or adjuvants in combatting antibiotic‐tolerant bacterial persisters, thereby providing promising avenues for future therapeutic strategies.

## 2 Results

### 2.1 MB6 Exhibits Antimicrobial Potency and Rescues *C. elegans* from MRSA Infection

**Figure 1:**
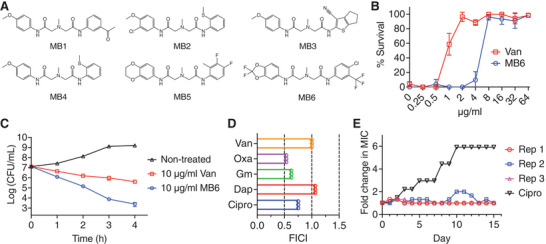
The methylazanediyl bisacetamide derivative MB6 exhibits antimicrobial potency with low probability for resistance development. A) The structures of methylazanediyl bisacetamide derivatives. B) MRSA‐infected *C. elegans glp‐4*(*bn2*);*sek‐1*(*km4*) animals were treated with the indicated concentrations of MB6 or vancomycin (Van) at 25 °C for 5 days. Following treatment, dead worms were stained with SYTOX Orange, and the surviving percentage of *C. elegans* animals was calculated for each well of the assay plate. Results are displayed as means ± SD (n = 3). C) Exponential‐phase *S. aureus* MW2 was exposed to MB6 or vancomycin for 4 h. The bacterial viability was assessed at hourly intervals, with a detection limit of 2 × 10^2^ CFU mL^−1^. Error bars represent SD (*n* = 3). D) Checkerboard microdilution assays were conducted using MB6 and ciprofloxacin (Cipro), daptomycin (Dap), gentamicin (Gm), oxacillin (Oxa), or vancomycin (Van) against *S. aureus* MW2. The fractional inhibitory concentration index (FICI) was calculated using the formula: FICI = MIC_A combined_/MIC_A alone_+ MIC_B combined_/MIC_B alone_. Interpretation of interaction: synergism (FICI ≤ 0.5), additive (0.5 < FICI ≤ 1). Individual data points are shown (*n* = 3). E) Emergence of spontaneous MB6‐ and ciprofloxacin (Cipro)‐resistant *S. aureus* MW2 mutants was monitored over 15 days of serial passage, conducted in triplicate (Rep 1, 2, and 3).

We screened an in‐house collection of synthetic and natural compounds for their ability to inhibit the growth of *S. aureus* MW2 growth as described in the Experimental Section. From this screen, we identified the synthetic small molecule 2‐[({[4‐chloro‐3‐(trifluoromethyl)phenyl]carbamoyl}methyl)(methyl)amino]‐N‐(2,2‐difluoro‐1,3‐dioxaindan‐5‐yl)acetamide (Enamine catalog no. Z46391767, hereafter referred to as MB6, **Figure** **1**A) that inhibits the growth MRSA strain MW2 at 100 µm. Among six commercially available methylazanediyl bisacetamide derivatives screened (Figure 1A), MB6 was the sole compound that inhibited MRSA growth. MB6 exhibited a minimum inhibitory concentration (MIC) of 4 µg mL^−1^ against MRSA MW2, whereas the derivatives MB1 to MB5 failed to inhibit MRSA MW2 growth even at 64 µg mL^−1^ (**Table** **1**).

**Table 1:** Antimicrobial activity of MB6 against ESKAPE strains.

Strains	MB1	MB2	MB3	MB4	MB5	MB6	Van	Gm	Cipro
*S. aureus* MW2	>64	>64	>64	>64	>64	4	1	0.5	0.25
*E. faecium* E007	>64	>64	>64	>64	>64	4	1	>64	64
*K. pneumoniae* WGLW2	>64	>64	>64	>64	>64	>64	>64	1	0.031
*A. baumannii* ATCC 17978	>64	>64	>64	>64	>64	>64	>64	1	0.25
*P. aeruginosa* PA14	>64	>64	>64	>64	>64	>64	>64	2	0.063
*E. aerogenes* ATCC 13048	>64	>64	>64	>64	>64	>64	>64	2	0.031

Abbreviations: Van, vancomycin; Gm, gentamicin; Cipro, ciprofloxacin.

To characterize the antimicrobial activity of MB6, we evaluated the ability of MB6 to inhibit the growth of five additional major bacterial pathogens (Table 1). MB6 displayed an MIC of 4 µg mL^−1^ against the Gram‐positive bacterium *Enterococcus faecium* E007 (Table 1). However, it exhibited no antimicrobial activity against Gram‐negative bacteria, including *Klebsiella pneumoniae* WGLW2, *Acinetobacter baumannii* ATCC17978, *Pseudomonas aeruginosa* PA14, and *Enterobacter aerogenes* ATCC13048 (Table 1). We further evaluated the antimicrobial potency of MB6 against a range of multidrug‐resistant *S. aureus* strains including MRSA clinical isolates, vancomycin‐intermediate *S. aureus* strains from the FDA‐CDC Antimicrobial Resistance Bank,^[^ ^13^ ^]^ and vancomycin‐resistant *S. aureus* strain VRS1.^[^ ^14^ ^]^ MB6 consistently showed MIC values of 4 µg mL^−1^ against these strains (**Table** **2**).

**Table 2:** Antimicrobial activity of MB6 against multidrug‐resistant *S. aureus*.

*S. aureus* strains	MB6	Oxa	Van	Dap	Gm	Cipro
ATCC 33591	4	>64	1	1	4	0.5
ATCC 43300	4	16	2	1	>64	0.5
HL16278	4	>64	1	2	0.5	1
HL17064	4	>64	1	2	>64	64
HL17078	4	64	1	2	64	16
HL18380	4	>64	1	2	0.5	16
HL18807	4	>64	1	2	0.5	64
HL18840	4	>64	1	2	>64	32
HL18883	4	>64	1	2	>64	>64
HL18888	4	>64	1	2	0.5	>64
HL20835	4	>64	1	2	0.5	32
HL21008	4	>64	1	1	0.5	0.125
VISA 0215	4	32	4	4	0.5	>64
VISA 0216	4	8	8	2	0.25	16
VISA 0217	4	8	4	4	1	32
VISA 0218	4	64	4	8	1	16
VISA 0219	4	>64	4	4	>64	16
VISA 0220	4	8	4	2	0.25	16
VISA 0221	4	8	4	8	>64	16
VISA 0222	4	0.25	4	2	1	0.25
VISA 0223	4	64	4	4	1	0.5
VISA 0224	4	>64	4	4	0.25	32
VISA 0225	4	16	4	16	0.5	32
VISA 0226	4	8	4	8	0.25	0.5
VISA 0227	4	1	4	4	0.5	64
VISA 0228	4	16	4	4	1	>64
VRS1	4	>64	>64	2	64	64

Abbreviations: Oxa, oxacillin; Van: vancomycin; Dap, daptomycin; Gm, gentamicin; Cipro, ciprofloxacin.

Since compounds with in vitro antimicrobial activity often exhibit limited in vivo effectiveness or toxicity,^[^ ^15^ ^]^ we assessed in vivo efficacy and toxicity of MB6 initially using a *Caenorhabditis elegans* infection assay.^[^ ^16^ ^]^ MB6 rescued *C. elegans* from MRSA‐mediated killing at a median effective concentration (EC_50_) of 5 µg mL^−1^, exhibiting no observable toxicity toward the nematodes at concentrations as high as 64 µg mL^−1^ (Figure 1B). The congruence between the EC_50_ and MIC values against MRSA MW2 (Figure 1B and Table 1) indicates that the effectiveness of MB6 in treating the MRSA infection in *C. elegans* is most likely attributable to its antimicrobial action.

### 2.2 MB6 Rapidly Kills Growing MRSA

We first sought to determine whether MB6 possesses bactericidal or bacteriostatic properties. Although MB6 has a higher MIC value of 4 µg mL^−1^ compared to the 1 µg mL^−1^ MIC value of vancomycin (Table 1), MB6 exhibited superior bactericidal potency (Figure 1C). At a concentration of 10 µg mL^−1^, MB6 led to an approximate 4‐log reduction in the viability of exponential‐phase *S. aureus* MW2 cells within 4 h (Figure 1C). Conversely, vancomycin, at 10 µg mL^−1^, resulted in only an approximate 1‐log reduction of viable MW2 cells (Figure 1C).

Next, we evaluated the inhibitory activity of MB6 against MRSA growth when combined with conventional antibiotics, applying a checkerboard method.^[^ ^17^ ^]^ As shown in Figure 1D, MB6 exhibited an additive effect with most of the tested antibiotics, including ciprofloxacin (FICI 0.75), gentamicin (FICI 0.63), oxacillin (FICI 0.53), and vancomycin (FICI 1). Interestingly, the combination of MB6 and daptomycin was not additive (FICI 1.06).

### 2.3 Lack of Detectable MB6 Resistance Development

We assessed the potential of *S. aureus* MW2 to develop resistance to MB6 by exposing the bacteria to serial passage in sub‐MIC levels of MB6 for 15 days. Ciprofloxacin was included as a positive control.^[^ ^18^ ^]^ Exposure to ciprofloxacin led to the emergence of *S. aureus* MW2 mutants exhibiting a sixfold increase in MIC to ciprofloxacin (Figure 1E). However, treatment with sub‐MIC MB6 for 15 days did not result in the generation of MB6‐resistant mutants (Figure 1E). Taken together, the results shown in Figure 1 demonstrate that MB6 is not only a fast‐killing agent against MRSA with a low propensity for resistance development, but also suggest potential for the combined use of MB6 with other commonly used antibiotics.

### 2.4 MB6 disrupts the membrane integrity of growing MRSA

**Figure 2:**
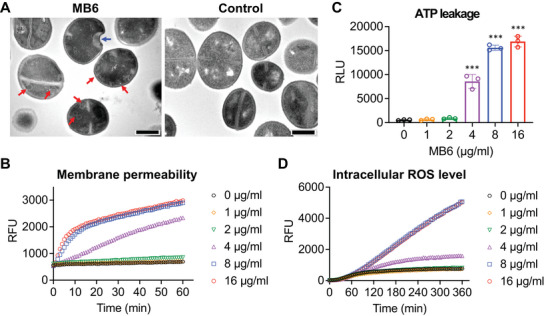
MB6 compromises the integrity of MRSA membranes and triggers the accumulation of intracellular ROS. A) Transmission electron micrographs illustrate mesosome‐like structures (red arrows) and a dent (a blue arrow) in growing MRSA cells subjected to 5× MIC MB6 treatment, compared to a 0.2% DMSO control. The scale bar in the lower right corner represents 1 µm. B) SYTOX Green uptake by growing *S. aureus* MW2 cells following MB6 treatment. Results are shown as means from triplicates. C) Cellular ATP leakage from growing MRSA cells exposed to MB6 for 1 h, assessed using an ATP luminescence assay. Individual data points are shown; error bars represent means ± SD (*n* = 3). Statistical differences were analyzed by one‐way ANOVA and post hoc Tukey test ^(****^ *p* < 0.0001). D) Intracellular ROS levels in growing *S. aureus* MW2 cells treated with MB6, determined using a fluorometric intracellular ROS kit. For panels (B) and (D), error bars (SD) are omitted for visual clarity. RFU and RLU mean relative fluorescence unit and relative luminescence unit, respectively.

We examined the effect of MB6 on the morphology of *S. aureus* MW2 through transmission electron microscopy. After 1 h of exposure to 20 µg mL^−1^ (5× MIC) MB6, *S. aureus* MW2 cells showed intracellular mesosome‐like structures and surface dents on the cell membrane (**Figure** **2**A), indicating that MB6 may target bacterial membranes. To delve into the effect of MB6 on bacterial membranes in more detail, we assessed the impact of MB6 on membrane integrity by employing the membrane‐impermeable DNA‐binding dye SYTOX Green.^[^ ^19^ ^]^ MB6 treatment resulted in a rapid increase in SYTOX Green fluorescence (Figure 2B) at MB6 concentrations equal to or higher than its MIC, suggesting that MB6 enhances bacterial permeability. Considering its rapid killing rate, the low probability for resistance development, and the rapid induction of SYTOX Green membrane permeabilization,^[^ ^20^ ^]^ we concluded that MB6 may operate as a membrane‐disrupting agent.

Membrane‐disrupting compounds can cause leakage of intracellular ATP.^[^ ^21^ ^]^ To ascertain if MB6 treatment results in ATP leakage from inside *S. aureus* cells, we employed a luciferin–luciferase bioluminescence assay. A significant increase in luminescence was observed in *S. aureus* MW2 cells treated with MB6 at concentrations of 4 µg mL^−1^ (1× MIC) or higher (Figure 2C), indicating MB6 causes membrane disruption, subsequently leading to the leakage of intracellular ATP. Moreover, previous studies have demonstrated that membrane disruption can trigger an intracellular buildup of reactive oxygen species (ROS).^[^ ^22^ ^]^ Consistent with these reported findings, we observed a surge in the level of intracellular ROS in *S. aureus* cells treated with 4 µg mL^−1^ MB6 or higher (Figure 2D). Collectively, these findings suggest that MB6 operates as a membrane‐disrupting agent, leading to the death of *S. aureus* cells.

### 2.5 MB6 Binds to and Embeds in the MRSA Lipid Bilayer

**Figure 3:**
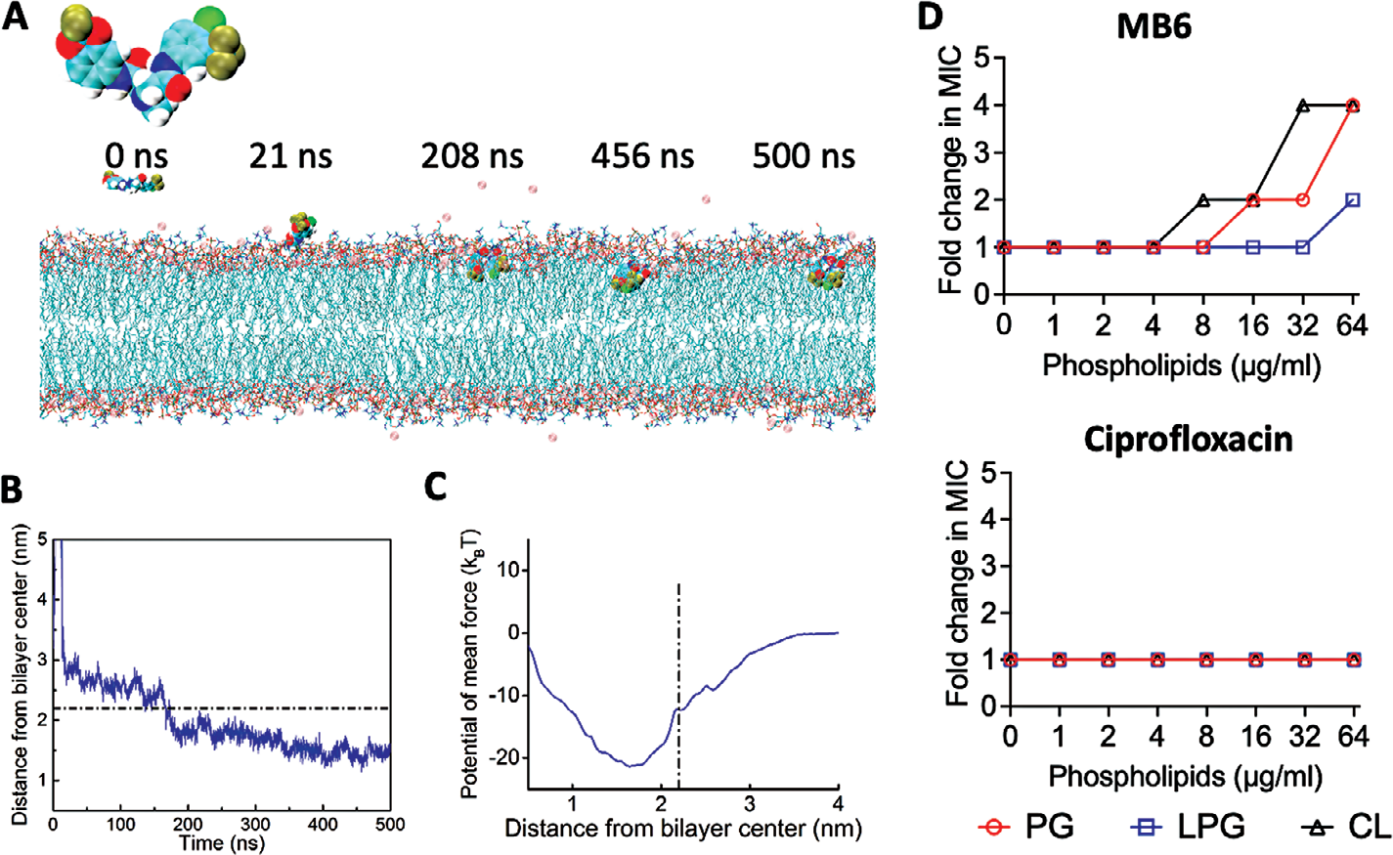
MB6 interacts with membrane phospholipid bilayers. A) Representative simulated configurations of MB6 interacting 7DOPC/3DOPG lipid bilayers including initial attachment, penetration, to equilibrium embedding. MB6 and sodium ions are depicted as large spheres; phospholipids are represented as chains. A magnified view of MB6 is also included. The atoms in MB6, phospholipids, and sodium ions are colored as follows: hydrogen (white), oxygen (red), nitrogen (blue), chlorine (green), fluorine (tan), carbon (cyan), phosphorus (orange), and sodium (pink). Water molecules are not shown for clarity. B) The representative temporal evolution of the Center of Mass (COM) distance between MB6 and the lipid bilayer throughout the simulated interaction process. C) The free energy profile of MB6 penetrating the lipid bilayer as a function of the COM distance of the bilayer. The dot‐dashed black lines in (B) and (C) represent the surface of the membrane. D) Change in MB6 MIC in the presence of phosphatidylglycerol (PG), lysyl phosphatidylglycerol (LPG), or cardiolipin (CL), evaluated using checkerboard microdilution assays. Phospholipid concentrations ranged from 0 to 64 µg mL^−1^. Ciprofloxacin was used as a negative control. The experiment was conducted in triplicate, with all replicates demonstrating consistent MIC changes.

We further investigated the membrane‐disrupting ability of MB6 at the molecular level using all‐atom molecular dynamics (MD) simulations based on the negatively charged bacterial membrane lipid bilayer model consisting of dioleoyl‐sn‐glycero‐3‐phosphocholine (DOPC) and 1,2‐dioleoyl‐sn‐glycero‐3phospho‐(1′‐rac‐glycerol) (DOPG) at a ratio of 7 to 3.^[^ ^8^, ^23^ ^]^ The MD simulations showed that MB6 is initially recruited to the lipid membrane surface due to the binding of its polar regions, which includes the central acetamide and terminal fluoro and chloro groups, with the hydrophilic heads of lipids via polar interactions (**Figure** **3**A; Video S1, Supporting Information). After ≈100 ns of persistent attachment, MB6 penetrates into the membrane interior, exhibiting a folded molecular configuration with two nonpolar benzene rings vertically embedded in the lipid bilayer, optimizing its hydrophobic interactions with the lipid tails. This embedded configuration remains stable throughout the rest of the simulation as indicated by temporal evolution of the center of mass (COM) distance between MB6 and the lipid bilayer (Figure 3A,B). Further, potential mean force calculations using the umbrella sampling method^[^ ^24^ ^]^ substantiated the conclusion that insertion of MB6 into the lipid bilayer is energetically favorable, exhibiting a transfer energy of ≈20*k_B_* T (Figure 3C).

To further understand the interaction between MB6 and the lipid bilayers of *S. aureus*, we explored the binding affinity of MB6 to key phospholipid constituents of the *S. aureus* membrane, namely phosphatidylglycerol (PG), lysyl‐phosphatidylglycerol (LPG), and cardiolipin (CL).^[^ ^25^ ^]^ The rationale was that if MB6 exhibits a specific affinity for any of these phospholipids, external supplementation with an excess of that phospholipid should attenuate the antimicrobial activity of MB6. For the negative control, we chose ciprofloxacin, which is known to traverse rather than to stably embed in membrane lipid bilayers.^[^ ^26^ ^]^ As expected, the antimicrobial potency of ciprofloxacin remained unaffected by the presence of any of the tested phospholipids (Figure 3D). In contrast, the MIC of MB6 increased in the presence of varying concentrations of either CL or PG in a dose‐dependent manner. Specifically, with CL concentrations of 8 and 32 µg mL^−1^, the MIC of MB6 increased by a factor of 2 and 4, respectively (Figure 3D). Similarly, with PG concentrations of 16 and 64 µg mL^−1^, the MIC of MB6 also increased by a factor of 2 and 4, respectively (Figure 3D). However, a high concentration of 64 µg mL^−1^ LPG resulted in only a modest 2‐fold increase in the MIC of MB6 (Figure 3D). These results suggest that MB6 has a discernible preference for binding to CL and PG over LPG. Taken together, these findings indicate that the antimicrobial activity of MB6 against *S. aureus* stems from its selective affinity for certain phospholipids, specifically CL and PG. Upon binding, MB6 integrates into the membrane lipid bilayers, instigating a disruption in membrane integrity, leading to an amplified intracellular ROS accumulation and ultimately resulting in bacterial cell death.

### 2.6 MB6 Displays Preferential Interaction with Bacterial Compared to Mammalian Membranes

**Figure 4:**
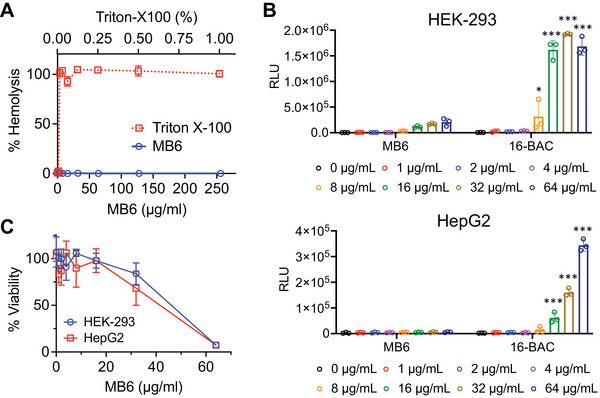
MB6 selectively targets bacterial membranes. A) Human erythrocytes (2%) were exposed to various concentrations of MB6 for 1 h at 37 °C. A sample treated with 1% Triton X‐100, known to induce complete hemolysis, was used as a reference. Results are presented as means ± SD (*n* = 3). B) ATP leakage from HEK‐293 or HepG2 cells, treated with a series of MB6 concentrations, was evaluated using an ATP luminescence assay. A cationic detergent 16‐BAC was used as a positive control. Individual data points are shown; error bars denote means ± SD (n = 3). Statistical differences were analyzed using two‐way ANOVA and post hoc Tukey test (^*^ *p* < 0.05 and ^****^ *p* < 0.0001). C) Viability of HEK‐293 and HepG2 cells was evaluated after treating with various concentrations of MB6 for 24 h. Cell viability was determined based on the conversion of WST‐1 dye to formazan by viable cells, detectable through absorbance readings at 450 nm. Results are presented as means ± SD (*n* = 3).

Given the critical challenge often faced in the development of membrane‐targeting antimicrobials, whereby such agents tend to interact indiscriminately with both bacterial and mammalian membranes,^[^ ^20^ ^]^ we examined the interaction of MB6 with various types of mammalian cell membranes. Initially, we tested MB6's effect on human erythrocytes and found no observable hemolysis up to a concentration of 256 µg mL^−1^ (**Figure** **4**A), indicating a selectivity index (median hemolytic concentration/MIC) of greater than 64 (Table S1, Supporting Information). We further validated this finding by evaluating ATP leakage from the human embryonic kidney cell line HEK‐293 and the human hepatoma cell line HepG2. No significant ATP leakage was observed when these cells were treated with MB6 up to 64 µg mL^−1^, whereas the cationic detergent benzyldimethylhexadecylammonium chloride (16‐BAC) induced ATP leakage from HEK‐293 and HepG2 cells at concentrations of 8 and 16 µg mL^−1^ or higher, respectively (Figure 4B). Additionally, cytotoxicity assessments of MB6 on HEK‐293 and HepG2 cells after 24‐h treatment showed median lethal concentrations (LC50) of 47 and 40 µg mL^−1^, respectively. This translates to a therapeutic index (LC50/MIC) of 10 or higher, suggesting that it would exhibit relatively low toxicity in vivo (Figure 4C, Table S1, Supporting Information).^[^ ^27^ ^]^ These collective observations indicate that MB6 displays a distinct selectivity for bacterial membranes over mammalian membranes. This observation is consistent with the absence of any detectable toxicity in the *C. elegans*‐MRSA infection assay (Figure 1B).

### 2.7 MB6 Shows Limited Bactericidal Activity Against MRSA Persisters

**Figure 5:**
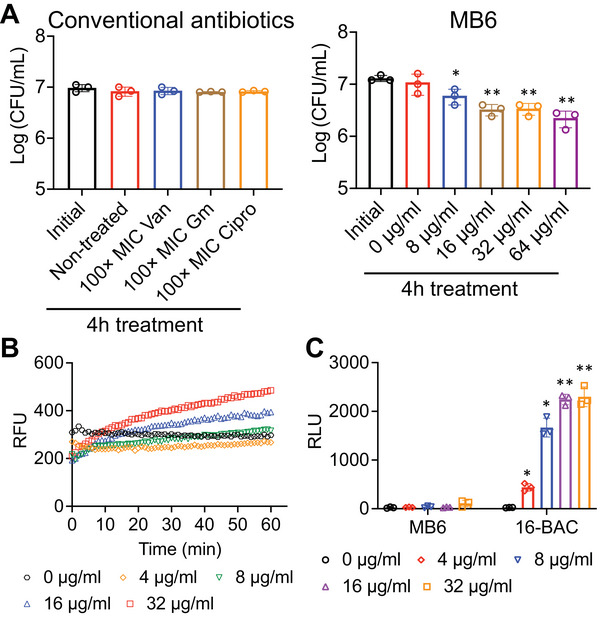
MB6 shows weak killing activity against MRSA persisters. A) MRSA MW2 persister cells were treated with 100× MIC conventional antibiotics or a range of concentration of MB6 for 4 h. Viability was measured by serial dilution and plating on agar plates. B) Uptake of SYTOX Green (Ex = 485 nm, Em = 525 nm) by MRSA MW2 persister cells treated with the indicated concentrations of MB6. Results are shown as means; *n* = 3 biologically independent samples. Error bars not shown for clarity. C) ATP leakage from MRSA MW2 persister cells treated with a range of concentrations of MB6 or 16‐BAC. For panels (A) and (C), individual data points are shown; error bars represent means ± SD (*n* = 3). Statistical differences were analyzed by one‐way ANOVA and post hoc Tukey test (^*^ *p* < 0.05, ^**^ *p* < 0.01, ^***^ *p* < 0.001).

We sought to evaluate the bactericidal activity of MB6 against MRSA persisters. Persister cells were produced by treating exponential‐phase *S. aureus* MW2 cells with 5 mm arsenate for 30 min, which is known to block ATP synthesis and to cause the formation of persister cells.^[^ ^4^ ^]^ The generation of MRSA persisters was confirmed by high levels of tolerance to 100× MIC of vancomycin, gentamicin, or ciprofloxacin, each of which employs different modes of antimicrobial activity (**Figure** **5**A, Figure S1A, Supporting Information). Somewhat unexpectedly, unlike its effect on growing MRSA cells (Figure 1C), MB6 showed limited effectiveness against MRSA persister cells (Figure 5A; Figure S1B, Supporting Information). A 4‐h exposure to MB6 at a concentration of 64 µg mL^−1^ resulted in ≈80% killing of MRSA persisters (Figure 5A).

To understand why the bactericidal potency of MB6 is diminished against persister cells, we assessed its capacity to disrupt the membrane of MRSA persisters using the SYTOX Green permeabilization assay. Whereas there was a significant decrease in the ability of MB6 to permeabilize persister cells compared to exponentially growing cells, MB6 still permeabilized the membrane of MRSA persisters at concentrations of 16 µg mL^−1^ or higher (Figure 5B), a concentration that is four times greater than what is required against growing MRSA cells (Figure 2B). Subsequently, we confirmed that arsenate‐induced persister cells still contain a detectable amount of intracellular ATP by treating with 16‐BAC as a positive control (Figure 5C). In contrast to the ability of MB6 to induce membrane permeability, no ATP leakage from MRSA persisters was observed, even upon treatment with MB6 up to 32 µg mL^−1^ (Figure 5C). These findings suggest that although MB6 inflicts sufficient membrane damage on MRSA persisters to allow SYTOX Green permeabilization, this level of damage is not sufficient to cause ATP leakage.

### 2.8 MB6 Potentiates the Bactericidal Activity of Gentamicin Against MRSA Persisters

**Figure 6:**
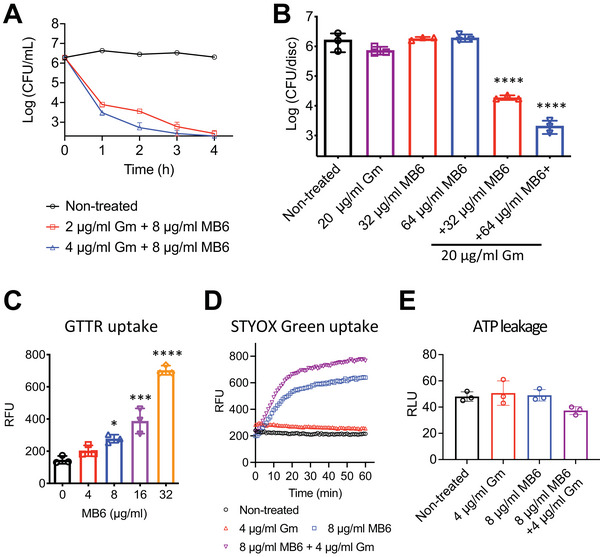
MB6 potentiates a bactericidal activity of gentamicin against MRSA persisters. A) Planktonic MRSA MW2 persisters were treated with the indicated concentrations of MB6 and gentamicin (Gm) combination. B) Biofilm MRSA MW2 persisters were treated similarly for 24 h. CFUs were measured by serial dilution and plating on CaMH agar plates. Data points on the *x*‐axis are below the level of detection (2 × 10^2^ CFU mL^−1^, or 2 × 10^2^ CFU per disc). Error bars represent mean ± SD (*n* = 3). C) Uptake of Texas Red‐gentamicin conjugates (Ex = 589 nm, Em = 615 nm) into MRSA MW2 persisters following treatment with indicated concentrations of MB6. Error bars denote mean ± SD. D) SYTOX Green uptake (Ex = 485 nm, Em = 525 nm) by MRSA MW2 persister cells treated with MB6, gentamicin (Gm), or their combination. Results are displayed as means; *n* = 3 biologically independent samples. Error bars are omitted for clarity. E) ATP leakage from MRSA MW2 persister cells post‐treatment with MB6, gentamicin (Gm), or their combination. Error bars denote means ± SD (*n* = 3). For panels (B), (C), and (E), individual data points represent biologically independent samples and statistical differences were analyzed by one‐way ANOVA and post hoc Tukey test (*p < 0.05, ***p < 0.001, ****p < 0.0001).

Whereas MB6 exhibits limited antimicrobial activity against MRSA persisters, it clearly increased the membrane permeability of these cells as determined by SYTOX Green uptake (Figure 5B). This observation led us to hypothesize that MB6 might promote the cellular uptake of aminoglycosides into MRSA persisters. Indeed, combining the aminoglycoside antibiotic gentamicin with MB6 led to a dose‐dependent eradication of MRSA persisters (**Figure** **6**A). Remarkably, a combination of just 4 µg mL^−1^ (8× MIC) of gentamicin with 8 µg mL^−1^ (2× MIC) MB6 completely eradicated ≈5×10^6^ CFU mL^−1^ MRSA persisters within a short 4‐h span (Figure 6A). In a time‐killing kinetic assay, synergism is defined as an equal to or greater than 2‐log decrease in CFU mL^−1^ by the antimicrobial combination compared to the most active constituent alone.^[^ ^17^ ^]^ Since 50 µg mL^−1^ (100× MIC) gentamicin and 64 µg mL^−1^ (16× MIC) MB6 used individually led to no reduction or less than a 1‐log reduction in the viability of MRSA persister cells, respectively (Figure 5A), these results demonstrate a synergistic killing effect of the combination of MB6 and gentamicin against MRSA persisters.

We next investigated whether MB6 could similarly enhance the efficacy of gentamicin against MRSA persisters within biofilms. These biofilm persister cells showed some tolerance to 20 µg mL^−1^ gentamicin or 64 µg mL^−1^ MB6 (Figure 6B). However, a combination of 20 µg mL^−1^ gentamicin with either 32 or 64 µg mL^−1^ MB6 resulted in an ≈2‐log or 3‐log decrease in biofilm viability, respectively (Figure 6B).

We proceeded to explore the underlying synergistic lethal mechanism between MB6 and gentamicin. First, we evaluated whether MB6 enhances the uptake of gentamicin into MRSA persisters using Texas Red‐conjugated gentamicin (GTTR). Notably, the uptake of GTTR into persister cells exhibited a significant MB6‐dose‐dependent increase ranging from 8 to 32 µg mL^−1^ (Figure 6C). We also examined whether gentamicin could augment the membrane activity of MB6. Although gentamicin did not permeabilize the membrane of MRSA persisters up to a concentration of 32 µg mL^−1^ (Figure S2A, Supporting Information), adding 4 µg mL^−1^ gentamicin to 8 µg mL^−1^ MB6 considerably enhanced the membrane permeability of these persister cells (Figure 6D). In contrast to gentamicin, vancomycin did not promote the ability of MB6 to permeabilize MRSA persister membranes (Figure S2B, Supporting Information).

Interestingly, even though gentamicin increased the membrane permeability of MB6, the combined treatment of MRSA persisters with gentamicin plus MB6 did not trigger ATP leakage (Figure 6E). This latter result indicates that the synergistic bactericidal effect of the MB6 and gentamicin combination could be attributed to the enhanced uptake of gentamicin rather than the increased extent of membrane damage induced by the combination treatment (Figure S3, Supporting Information).

### 2.9 MB6 Amplifies the Lethal Activity of Daptomycin Against MRSA Persisters

**Figure 7:**
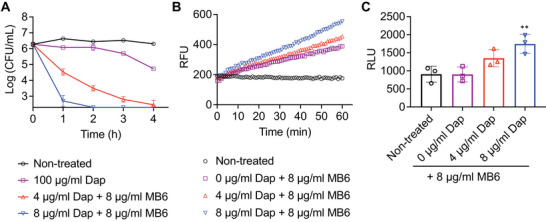
MB6 enhances bactericidal activity of daptomycin against MRSA persisters. MRSA MW2 persisters were treated with the indicated concentrations of daptomycin (Dap) alone or in combination with MB6. CFUs were measured by serial dilution and plating on CaMH agar plates. The data points on the *x*‐axis are below the level of detection of 2 × 10^2^ CFU mL^−1^. Error bars represent means ± SD (*n* = 3). B) Uptake of SYTOX Green (Ex = 485 nm, Em = 525 nm) by MRSA MW2 persister cells treated with the indicated concentrations of MB6, alone or in combination with daptomycin (Dap). Results are shown as means (*n* = 3) Error bars are omitted for clarity. C) ATP leakage from MRSA MW2 persister cells after treatment with MB6 alone or in combination with daptomycin (Dap). Individual data points are shown; error bars denote means ± SD (*n* = 3). Statistical differences were analyzed by one‐way ANOVA and post hoc Tukey test (^**^ *p* < 0.01).

We also examined whether MB6 exhibits synergism with daptomycin against MRSA persisters. While daptomycin alone at a concentration of 100 µg mL^−1^ (100× MIC) only resulted in approximately a 1‐log decrease in persister viability, its effectiveness significantly increased when combined with 8 µg mL^−1^ (2× MIC) MB6 (**Figure** **7**A). Specifically, when combined with 8 µg mL^−1^ MB6, 4 µg mL^−1^ (4× MIC) or 8 µg mL^−1^ (8× MIC) of daptomycin completely eradicated ≈5×10^6^ CFU mL^−1^ MRSA persisters within 4 and 2 h, respectively (Figure 7A). This combination resulted in a greater than 2‐log reduction in bacterial viability compared to the results of 100× MIC daptomycin and 64 µg mL^−1^ MB6 when used individually (Figures 5A and 7A), indicating synergism between MB6 and daptomycin against MRSA persisters.

Given that the bactericidal activity of both MB6 and daptomycin is derived from their ability to disrupt bacterial membranes, we assessed whether MB6 could enhance the membrane‐disrupting activity of daptomycin against persister cells. Daptomycin alone failed to induce either SYTOX Green membrane permeabilization or ATP leakage from MRSA persisters (Figure S4A,B, Supporting Information). However, when combined with 8 µg mL^−1^ MB6, daptomycin induced rapid membrane permeabilization in a dose‐dependent manner (Figure 7B). Similarly, the combination of daptomycin and 8 µg mL^−1^ MB6 caused ATP leakage from MRSA persister cells, also in a dose‐dependent manner (Figure 7C). These results suggest that while daptomycin alone cannot induce substantial damage to intact persister membranes, when the persister membrane is initially disrupted by MB6, daptomycin can cause lethal membrane disruption, significant enough to induce the leakage of intracellular ATP in bacterial persisters (Figure S3, Supporting Information). Thus, the synergism between these agents is likely attributed to the ability of MB6 to make the persister membrane more vulnerable to daptomycin.

### 2.10 Three‐Way Combination Of Gentamicin, Daptomycin, and MB6 is Highly Efficacious Against MRSA Persisters

**Figure 8:**
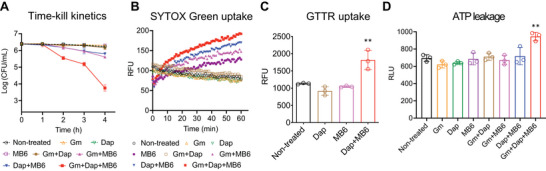
MB6 potentiates anti‐persister activity of daptomycin and gentamicin combination against MRSA persisters. A) MRSA MW2 persisters were treated with 2×MIC gentamicin (Gm), daptomycin (Dap), MB6, or their respective double or triple combinations. CFUs were measured by serial dilution and plating on CaMH agar plates. Error bars indicate means ± SD (*n* = 3). B) SYTOX Green uptake (Ex = 485 nm, Em = 525 nm) was assessed in MRSA MW2 persister cells following treatment with 2×MIC gentamicin (Gm), daptomycin (Dap), MB6, or their respective double or triple combinations. Results are displayed as means (*n* = 3); error bars have been omitted for clarity. C) The uptake of Texas Red‐gentamicin conjugates (Ex = 589 nm, Em = 615 nm) was evaluated in MRSA MW2 persisters after treatment with 2×MIC daptomycin (Dap), MB6, or their combination. D) ATP leakage from MRSA MW2 persister cells was observed post‐treatment with 2×MIC gentamicin (Gm), daptomycin (Dap), MB6, or their double‐or‐triple combinations. For panels (C) and (D), individual data points are displayed; error bars represent mean ± SD (*n* = 3). Statistical differences were calculated using a one‐way ANOVA followed by a post hoc Tukey test (^**^ *p* < 0.01).

Based on our findings that a combination of MB6 and daptomycin enhances membrane permeabilization in MRSA persister cells (Figure 7B), we hypothesized that the combination of MB6 + daptomycin might facilitate gentamicin uptake more than either MB6 or daptomycin alone. We tested this by treating MRSA persister cells with double or triple combinations of MB6, daptomycin, and gentamicin, each at 2× MIC. Single treatments using 2× MIC of MB6, daptomycin, or gentamicin, as well as the daptomycin + gentamicin combination, did not significantly alter MRSA persister viability (**Figure** **8**A). Moreover, a combination of daptomycin and gentamicin each at 10× MIC resulted in only a 1‐log reduction in persister viability (Figure S5A, Supporting Information). Consistent with the data in the preceding section, pairing 2× MIC (8 µg mL^−1^) MB6 with either 2× MIC (2 µg mL^−1^) daptomycin or 2× MIC (1 µg mL^−1^) gentamicin caused a reduction in viability of MRSA persisters ranging from 0.6 to 0.8‐log (Figure 8A). Most strikingly, when MB6, daptomycin, and gentamicin were jointly used at 2× MIC, a 3‐log decrease in MRSA persister viability was observed. This surpasses a 2‐log reduction when compared to any of the single or double treatments (Figure 8A)—an effect that indicates synergistic lethal activity.^[^ ^17^ ^]^

We further investigated if the observed synergistic action of MB6 with the gentamicin‐daptomycin combination was a unique property of MB6. For this, we tested the membrane‐active antimicrobials adarotene and PQ401, both known for their ability to permeabilize membranes but with limited lethality against MRSA persisters.^[^ ^8^, ^28^ ^]^ Unlike MB6, adarotene at 2× MIC augmented the bactericidal action of gentamicin but failed to boost the efficacy of the gentamicin and daptomycin combination against MRSA persisters (Figure S5B, Supporting Information). Similarly, PQ401 at 2× MIC did not intensify the effectiveness of gentamicin, daptomycin or gentamicin + daptomycin on persister cells (Figure S5B, Supporting Information). These results highlight the distinct activity of MB6, acting as a potent adjuvant with the gentamicin‐daptomycin combination against MRSA persisters, even at concentrations as low as 2× MIC.

We then sought to unravel the mechanisms underpinning this triple synergy. As expected, the triple combination of MB6 + daptomycin + gentamicin each at 2× MIC markedly increased SYTOX Green membrane permeabilization in MRSA persisters compared to the daptomycin + gentamicin combination at 2× MIC (Figure 8B). Furthermore, a combined treatment of 2× MIC MB6 and 2× MIC daptomycin led to a significant uptake of GTTR into persisters, an effect not observed with either 2× MIC MB6 or 2× MIC daptomycin alone (Figure 8C). Intriguingly, the triple combination of MB6, daptomycin, and gentamicin, each at 2× MIC, resulted in a marked intracellular ATP leakage from MRSA persister cells, a phenomenon not seen with any of the single or double combinations at 2× MIC (Figure 8D). These findings suggest that MB6, daptomycin, and gentamicin may work synergistically to disrupt MRSA persister membranes, facilitating both gentamicin uptake and triggering the leakage of intracellular ATP (Figure S3, Supporting Information).

## 3 Discussion

Infections involving *S. aureus* persisters are often recurrent and difficult to manage with current antibiotic chemotherapy. Leveraging antibiotic synergism could offer a promising strategy to combat bacterial persisters as this approach allows for reduced dose‐dependent toxicity while augmenting bactericidal activity. In this study, we identified a novel membrane‐active compound, MB6, with a methylazanediyl bisacetamide structure, that exhibits antimicrobial potency against multidrug‐resistant *S. aureus*. Key features of MB6 include its rapid killing kinetics, low propensity for resistance development, and preferential targeting of Gram‐positive bacterial membranes over mammalian membranes. Importantly, MB6 acts as a potent enhancer for gentamicin, daptomycin, and a combination of gentamicin and daptomycin, enabling significant reductions in effective concentrations for eradicating MRSA persisters.

Although MB6 is a membrane‐disrupting antimicrobial, it was ineffective in killing MRSA persisters. Our group and others have previously demonstrated that not all membrane‐disrupting antimicrobial agents have bactericidal activity against persister cells.^[^ ^4^, ^8^, ^28^, ^29^ ^]^ For instance, whereas CD437, bithionol, and nTZDpa can eradicate MRSA persisters, other membrane‐active agents, such as MB6 as well as adarotene, PQ401, and rhamnolipids, have limited standalone anti‐persister activity.^[^ ^4^, ^8^, ^28^, ^29^ ^]^ Furthermore, minor structural modifications in CD437, bithionol, or nTZDpa can nullify their bactericidal activity against MRSA persisters, even though the modified analogs retain the ability to induce membrane permeabilization.^[^ ^8^, ^29^ ^]^ The results suggest that the membranes of MRSA persisters are more robust compared to those in growing cells. Thus, only membrane‐active antimicrobials that can inflict sufficiently severe damage may effectively eradicate MRSA persisters.

The extent of membrane damage in MRSA persisters has been assessed using SYTOX Green membrane permeability, membrane fluidity measured by Laurdan generalized polarization, and intracellular ATP leakage detected by luciferase‐based bioluminescence.^[^ ^8^, ^21^, ^30^ ^]^ Although SYTOX Green was originally developed as a dead cell detection dye,^[^ ^19^ ^]^ some antimicrobials such as MB6, adarotene, and PQ401, which induce rapid SYTOX Green membrane permeabilization, fail to eradicate MRSA persister cells. This shows that measuring SYTOX Green membrane permeability by itself may not be sufficient for estimating a compound's ability to kill persister cells. In addition, for some membrane‐disrupting compounds, alterations in membrane fluidity detected by Laurdan dye correlate with the level of membrane damage required to kill MRSA persisters. However, Laurdan dye has drawbacks, such as poor photostability and high rates of internalization,^[^ ^31^ ^]^ thereby limiting its use. We also observed that MB6 directly interacts with Laurdan dye, interfering with the assessment of changes in membrane fluidity. Therefore, the rapid assessment of intracellular ATP leakage may serve as a more reliable indicator for determining the degree of membrane damage to induce the death of bacterial persister cells.

Our findings suggest that the synergistic effects observed between MB6 + gentamicin and between MB6 + gentamicin + daptomycin against MRSA persisters can be attributed to amplified membrane permeabilization and a consequent increase in gentamicin uptake (Figure S3, Supporting Information). Individually, gentamicin does not rapidly permeabilize the membrane of MRSA persisters (Figure S2, Supporting Information). Yet, when the integrity of these membranes is compromised by MB6, gentamicin likely not only diffuses through but also further intensifies the membrane permeabilization process (Figure 6D). This indicates that the gentamicin influx into persister cells is hastened by its role in augmenting membrane permeability (Figure 6C). Intriguingly, however, the combinational action of MB6 and gentamicin on the persister membrane does not seem to be the primary cause for persister cell death, as their joint treatment does not lead to intracellular ATP leakage (Figure 6E). Rather, it is the elevated intracellular concentrations of gentamicin (Figure 6C), which inhibits protein synthesis, that appears to be the pivotal factor. This subtle interplay between MB6 and gentamicin provides a deeper understanding of their combined potency and points toward novel therapeutic approaches against MRSA persisters.

In our experimental settings, we noted discrepancies between antimicrobial interactions derived from checkerboard assays and those from time‐kill kinetic assays. The checkerboard assay evaluates growth inhibitory activity, mainly against growing MRSA cells, whereas the time‐kill kinetic assay assesses bactericidal activity against MRSA persister cells. Given this distinction, it is highly plausible that antimicrobial combinations demonstrating synergistic growth inhibition in a checkerboard assay might not exhibit equivalent synergistic bactericidal activity against MRSA persisters, and vice versa. For instance, in actively growing bacterial cells where the PMF is maintained,^[^ ^5^, ^6^ ^]^ gentamicin can enter these cells. MB6 leads to an increase in membrane permeability, which further promotes gentamicin uptake. This is most likely observed as an additive interaction in the checkerboard assay. In contrast, with the PMF being highly diminished in persister cells,^[^ ^4^, ^5^ ^]^ gentamicin uptake into these cells hinges on the membrane disruption caused by MB6. Following this initial disruption by MB6, gentamicin further amplifies its own uptake by enhancing the permeabilization of the already compromised membranes. This interaction is likely identified as synergism in a time‐kill kinetic assay. Overall, these contrasting results highlight the importance of considering the metabolic states of bacterial cells when assessing the synergistic capabilities of antimicrobial combinations.

Unlike gentamicin, the synergistic bactericidal mechanism between MB6 and daptomycin appears to result from reciprocal enhancement of membrane‐disrupting activity. Their combined effect not only leads to increased membrane permeabilization but also triggers significant intracellular ATP leakage (Figure 7B,C). Daptomycin is known to interact with PG,^[^ ^32^ ^]^ while CL prevents its penetration and membrane disruption.^[^ ^11^, ^33^ ^]^ In contrast, MB6 preferentially binds to CL, followed by PG (Figure 3E). It is, therefore, conceivable that MB6 binding to CL may pave the way for enhanced interaction between daptomycin and PG. Supporting this conclusion, the combination of daptomycin with MB6 results in both a pronounced SYTOX Green membrane permeability and elevated ATP leakage in MRSA persisters (Figure 7B,C), effects not observed when daptomycin was used alone (Figure S4A,B, Supporting Information). These results demonstrate that CL‐binding membrane‐active antimicrobials, like MB6, could serve as promising adjuvants to enhance daptomycin's potency against MRSA persisters. Nevertheless, more in‐depth research is needed to validate these initial observations and to further unravel the intricate synergistic mechanisms involved.

MB6 also potentiates the combination of gentamicin and daptomycin against MRSA persisters. Notably, a triple combination of each, at only 2× MIC, is sufficient to eradicate MRSA persisters, indicating significant dose‐sparing effects (Figure 8A). The mechanisms underlying this synergism involve enhanced membrane disruption, leading to increased membrane permeabilization, ATP leakage, and augmented cellular uptake of gentamicin. MB6 appears to play a critical role in this triple synergism, as the combination of gentamicin plus daptomycin alone does not induce either membrane permeabilization or intracellular ATP leakage in MRSA persisters (Figure 8B). Similarly, combinations of two membrane‐disrupting antimicrobials, like antimicrobial polymers and colistin, have been observed to synergistically enhance the antimicrobial activity of doxycycline against *P. aeruginosa*, a Gram‐negative pathogen, including multidrug‐resistant *P. aeruginosa* strains.^[^ ^34^ ^]^ Given that doxycycline, a protein synthesis inhibitor, relies on the transmembrane proton gradient for uptake, one of the elements of the PMF,^[^ ^35^ ^]^ this synergistic effect is likely attributable to enhanced membrane disruption by the combined action of antimicrobial polymers and colistin, which facilitates an increased influx of doxycycline. These observations imply that the use of dual membrane‐active agents could be an effective approach in developing combination therapies against multidrug‐resistant and ‐tolerant bacteria.

Previous research has shown that daptomycin can act synergistically with gentamicin. Their synergistic mechanism is thought to be due to daptomycin's ability to disrupt membranes, which in turn facilitates gentamicin uptake.^[^ ^36^ ^]^ However, this synergistic efficacy is not consistent across different strains and infection models.^[^ ^37^ ^]^ In our study, we found that daptomycin could neither permeabilize MRSA persister membranes nor exhibit synergism with gentamicin, presumably due to insufficient membrane damage inflicted by daptomycin on MRSA persisters. In contrast, MB6 appears to induce significant damage to the persister membranes, making them more susceptible to disruption by daptomycin, gentamicin, or their combination. Despite their cytotoxicity, gentamicin and daptomycin are employed in the treatment of severe *S. aureus* infections such as persistent bacteremia and infective endocarditis.^[^ ^38^ ^]^ In this context, the adjuvant potential of MB6 could be invaluable for not only enhancing the efficacy of current combination antimicrobial therapies but also reducing toxicity.

MD simulations predict that the polar branch group of the central acetamide, along with the terminal halogen groups in MB6, enables firm binding to hydrophilic lipid heads via polar interactions. This is consistent with our observations of other methylazanediyl bisacetamide compounds (Figure 1A), which, in the absence of halogen moieties or with only one side halogenated ring, do not exhibit antimicrobial activity. Interestingly, the role of terminal halogens in membrane attachments aligns with those observed in other membrane‐active antimicrobial agents, such as nTZDp, bithionol, and PQ401 that have been studied in our laboratories.^[^ ^8^, ^28^, ^29^ ^]^ Structure‐activity relationship (SAR) studies of nTZDpa and bithionol revealed that fluorine, when attached to an aromatic ring group, hinders deeper penetration into the membrane but provides strong attachment to the membrane surface, which results in a relatively lower antimicrobial activity compared to chlorine or bromine.^[^ ^8^, ^29^ ^]^ Conversely, larger halogens like chlorine or bromines provide relatively looser yet still firm binding to the membrane surface, while facilitating deep penetration and severe distortion within membranes.^[^ ^8^, ^29^ ^]^ Thus, we speculate that the antimicrobial or adjuvant activity of MB6 could be further optimized by altering its halogen moieties.

It is worth noting that in this study, we employed the widely used, albeit simplified, atomistic *S. aureus* model,^[^ ^8^, ^23^ ^]^ capable of capturing the primary interactions between MB6 and the bacterial membrane, despite not explicitly accounting for CL lipids. Nevertheless, our phospholipid‐binding assay revealed a marked preference of MB6 for CL lipids, thus highlighting the necessity for the employment and further development of a more sophisticated and realistic model.^[^ ^8^, ^39^ ^]^ Such a model is indispensable in establishing a comprehensive simulation platform with the potential to expedite the process of antibiotic discovery, especially considering the significant potential of membrane‐active agents.^[^ ^15^, ^20^, ^40^ ^]^

## 4 Conclusion

In conclusion, this study provides evidence of the membrane‐active antimicrobial and adjuvant potency of a specific methylazanediyl bisacetamide, MB6, against multidrug‐resistant and ‐tolerant MRSA. We illuminate its mode of action and reveal synergistic effects when combined with gentamicin, daptomycin, or both, thereby setting the stage for innovative therapeutic strategies against persistent MRSA infections. These findings highlight the promise of MB6 and similar compounds in combating the resilience of MRSA persister cells, but further investigations are required to evaluate their in vivo efficacy and potential cytotoxicity to ensure their safety and effectiveness for clinical application.

## 5 Experimental Section

### Key Resources and Agents

Key resources and agents, including bacterial strains, chemicals, dyes, and kits used in this study are listed in Table S2 (Supporting Information).

### Bacterial Strains and Growth Conditions

*S. aureus* was cultured in tryptic soy broth (TSB) and *Enterococcus faecium* was cultivated in brain heart infusion broth (BHI, Table S2). *Klebsiella pneumoniae*, *Acinetobacter baumannii*, *Pseudomonas aeruginosa*, and *Enterobacter aerogenes* were cultured in Lennox (LB) broth. To prepare overnight cultures, a single colony of each bacterial strain was inoculated into 5 mL of the appropriate broth and incubated at 37 °C for 18–24 h with constant shaking at 200 rpm.

### Antimicrobial Compound Screen

An in‐house collection of ≈2000 synthetic and natural compounds, housed in the College of Pharmacy at Ewha Womans University, was screened for antimicrobial activity. These compounds were either sourced from commercial suppliers, were synthesized in‐house, or were extracted from natural products by researchers within the College. Each compound in the collection was dissolved in DMSO to concentrations of 10 mm or 10 mg mL^−1^. An overnight culture of *S. aureus* MW2 was diluted to ≈1×10^6^ CFU mL^−1^ in cation‐adjusted Mueller–Hinton (CaMH) Broth. Thereafter, 50 µL of this diluted culture was introduced into 96‐well assay plates (Corning Falcon cat no 353072, USA), with each well containing 50 µL of compounds diluted in CaMH to the final concentrations of 100 µm or 64 µg mL^−1^. The assay plates were incubated for 20 h at 37 °C. Subsequently, the optical density at 600 nm (OD_600_) was measured using a Cytation 5 multi‐mode reader (BioTek, USA). Growth inhibition was defined at OD_600_ < 0.1. The screen was conducted in duplicate.

### Preparation of Antimicrobial and Chemical Solutions

Oxacillin, gentamicin, vancomycin, and daptomycin were dissolved in double‐distilled water (ddH_2_O), while ciprofloxacin was dissolved in 0.1 n HCl. The six methylazanediyl bisacetamide derivatives, each with a purity of >90%, were sourced from Enamine Ltd (Cat no. Z46395311, Z46377505, Z46395636, Z46377518, Z46384180, Z46391767; Kyiv, Ukraine) and along with adarotene and PQ401, were dissolved in dimethyl sulfoxide (DMSO). All antimicrobial solutions were initially prepared as stock solutions at 10 or 5 mg mL^−1^. For various experimental assays, these stock solutions were further diluted as needed in the appropriate media or buffers to obtain the desired working concentrations. All assays in which daptomycin was tested had a CaCl_2_ supplement, with a final concentration of 50 µg mL^−1^.

### Determination of Minimum Inhibitory Concentration (MIC)

The MICs of antimicrobial agents were determined following the microbroth dilution method in accordance with the guidelines provided by Clinical and Laboratory Standards Institute.^[^ ^41^ ^]^ Briefly, overnight bacterial cultures except for *Enterococcus* spp. were diluted to a concentration of 1×10^6^ CFU mL^−1^ using CaMH Broth. *Enterococcus spp*. were diluted to the same concentration using BHI. The test compounds were then subjected to twofold serial dilutions with CaMH or BHI broth, resulting in a concentration range spanning from 128 to 0.25 µg mL^−1^ in 96‐well assay plates (Corning Falcon cat no 353072, USA). Subsequently, 50 µL of the diluted bacterial samples were added to the wells containing 50 µL of the media with dissolved test compounds at varying concentrations. A non‐treated sample was employed as a negative control. The assay plates were incubated overnight at 37 °C, after which the optical density at 600 nm (OD_600_) was measured using a Cytation 5 multi‐mode reader (BioTek, USA). Bacterial growth was defined at OD_600_ ≥ 0.1. The experiments were independently replicated three times.

### *C. elegans*‐MRSA Infection Assay

The *C. elegans*‐MRSA infection assay was conducted following a previously described methodology with minor modifications.^[^ ^28^ ^]^ The temperature‐sensitive sterile and immunocompromised *C. elegans* strain AU37 [*glp‐4*(*bn2*);*sek‐1*(*km4*)] was grown to gravid adults on *Escherichia coli* HB101 lawns on 10‐cm slow‐kill (SK) agar plates. Eggs were collected by bleach treatment and washed three times with M9 buffer. The eggs, resuspended in M9 buffer, were allowed to hatch into L1 larvae by gentle rocking at 15 °C for 48 h. Approximately 4500 L1 larvae were dispensed on each SK plate overlaid with an *E. coli* HB101 lawn and incubated at 25 °C for 52 h, during which the larvae grew into sterile young adults. The adult worms were harvested, washed six times, and resuspended in M9 buffer at a concentration of 1000 worms per 1 mL.

A black, clear‐bottom 384‐well plate (Corning no. 3712, USA) was loaded with 20 µL M9 buffer containing the desired concentrations of MB6 or vancomycin. Following this, 15 µL of the worm suspension was dispensed into each well of the 384‐well plate using a Multidrop Combi Reagent Dispenser (ThermoFisher, USA), yielding ≈15 worms in a well. A culture of *S. aureus* MW2 was diluted to OD_600_ 0.08 with 20% TSB in M9 buffer. Thirty‐five micoliters of the diluted culture was then added to the 384‐well assay plate, which was subsequently sealed with a gas‐permeable membrane (Breathe‐Easy; Diversified Biotech, USA). The assay plate was incubated in a humidified chamber at 25 °C for a period of 5 days.

Upon completion of the incubation, bacterial supernatant and biofilms in each well were eliminated by washing nine times with M9 buffer using a 405 TS microplate washer (BioTek, USA). To ensure comprehensive removal, the plate was agitated after every third wash cycle at 1800 rpm for 45 s using a MixMate (Eppendorf, Germany). The washed worms were then stained overnight with 0.7 µm SYTOX Orange, a stain selectively marking dead worms. Worms in each well were imaged using a Cytation 5 Cell Imaging Multi‐Mode Reader (BioTek, USA), capturing both transmitted light and RFP (excitation 531/40 nm, emission 593/40 nm) fluorescent images with a 4× objective. The infection assay was independently conducted three times.

### Exponential‐Phase MRSA Killing Kinetic Assay

An overnight culture of *S. aureus* MW2 was diluted 1:10 000 into 25 mL TBS in a 250‐mL flask. This diluted culture was incubated at 37 °C with 200 rpm shaking until reaching the exponential phase, signified by an OD_600_ of 0.1. The exponential‐phase cultures were then combined with an equal volume of prewarmed TSB containing twice the desired concentrations of each specific antimicrobial agent. Post‐incubation at 37 °C with shaking at 200 rpm, samples were collected every hour, washed once with phosphate buffered saline (PBS), subjected to tenfold serial dilutions with PBS, and were spot‐plated on CaMH agar plates. After an overnight incubation at 37 °C, viable cells were quantified by colony counting. The experiments were executed in biological triplicates.

### Resistance Selection via Serial Passage Assay

The selection of resistance was carried out via a 15‐day serial passage assay, which was performed with minor modifications to a previously established protocol.^[^ ^18^ ^]^ In brief, an expanded range of MB6 concentrations was generated through a twofold serial dilution of CaMH broth, beginning from initial MB6 concentrations of 20, 24, and 32 µg mL^−1^. This process was conducted across three columns of a 96‐well plate, creating 24 distinct concentration gradients. The plate contained three sets of the extended MB6 gradient, along with one set of an extended gradient of ciprofloxacin, used as a positive control. Next, 50 µL of *S. aureus* MW2 cells at a density of 10^6^ CFU mL^−1^ in CaMH were added to the 96‐well plate, which already contained 50 µL of the extended gradient of either MB6 or ciprofloxacin. The assay plate was then incubated at 37 °C for 24 h. The optical density at 600 nm was measured using a Cytation 5 multi‐mode reader (BioTek, USA), with bacterial growth defined at an OD_600_ ≥ 0.1. The sample that managed to grow at the highest antimicrobial concentration was then diluted 1000‐fold with CaMH, and transferred to a fresh extended gradient plate for the next passage. The remainder of the sample was mixed with glycerol to a final glycerol concentration of 16% and subsequently stored at −80 °C.

### Transmission Electron Microscopy

An overnight culture of MRSA MW2 was diluted 1:10 000 in fresh TSB, and incubated at 37 °C, 200 rpm until it reached exponential‐phase as indicated by an OD_600_ of 0.1. These exponential‐phase cells were treated with either 20 µg mL^−1^ (5× MIC) MB6 or 0.1% of DMSO (control) for 2 h at 37 °C with shaking at 200 rpm. The treated bacterial cells were washed twice with PBS and subsequently fixed in 1 mL of 0.1 m phosphate buffer (pH 7.4) including 2.5% glutaraldehyde for 2 h. Following this, the fixed bacterial cells were washed three times in 0.1 m phosphate buffer, and then fixed again with 1% osmium tetroxide in 0.1 m phosphate buffer (pH 7.4) for an hour, followed by two additional washes. The fixed cells then underwent a dehydration in a graded ethanol series (50%, 60%, 70%, 80%, 90%, 95%, 100%) for 10 min at each concentration, with a final two rounds in 100% ethanol. This was followed by 1‐h incubation in propylene oxide. Afterward, the cells were embedded in Epon‐812epoxy resin. Ultrathin sections, ≈60–70 nm thick, were prepared using a Leica EM UC7 ultramicrotome equipped with a diamond knife. The sections were stained with uranyl acetate and lead citrate. The samples were examined at the Ewha Medical Research Institute, using a Hitachi H‐7650 Transmission Electron Microscope (Tokyo, Japan), which was operating at an accelerating voltage of 80 kV. The images obtained represent the bacterial ultrastructure observed in more than ten individual bacterial cells.

### Membrane Permeability Assessment

The membrane permeability of MRSA was evaluated using the membrane‐impermeable DNA‐binding dye SYTOX Green, following a previously outlined procedure.^[^ ^8a^ ^]^ MRSA exponential‐phase or persister cells were washed three times with PBS and then adjusted to an OD_600_ of 0.4. Following this, SYTOX Green was added to the washed cells at a final concentration of 5 µm. The samples were incubated for 30 min at room temperature in the dark. A 50 µL aliquot from each sample was then dispensed into each well of a black 96‐well plate (Greiner Bio‐One Cat no. 665090), each well containing the indicated concentrations of the compounds. Fluorescence measurements were carried out at room temperature using a Cytation 5 multimode plate reader (BioTek, USA), set at excitation and emission wavelengths of 485 and 525 nm, respectively. Experiments were conducted in triplicates.

### Evaluation of ATP Leakage from Bacterial Cells

The release of extracellular ATP from both exponential‐phase and persister *S. aureus* MW2 cells was measured using the RealTime‐GloTM Extracellular ATP Assay (Promega, Madison, WI, USA). The 4× ATP assay kit reagent mixture was prepared according to the manufacturer's instructions. Cells were washed three times with PBS and then resuspended in PBS to adjust to an OD_600_ of 0.4. A 50 µL aliquot of this cell suspension was added to each well of a black, clear‐bottom 96‐well plate that had been pre‐filled with 50 µL of the antimicrobial agent at twice the final desired concentration. The plate was then incubated at 37 °C without shaking for an hour. Following this, 33.4 µL of the 4× reagent mixture was added to each well, and luminescence was quantified using a Cytation 5 multimode plate reader (BioTek, USA). All experiments were conducted in triplicate.

### Assessment of Intracellular Reactive Oxygen Species (ROS) Levels

The induction of intracellular ROS by MB6 in *S. aureus* MW2 was evaluated using the Fluorometric Intracellular ROS kit (Sigma–Aldrich, St. Louis, MO, USA), in accordance with the manufacturer's instructions. The bacteria were cultivated to reach an OD_600_ of ≈1.0 in the CaMH broth. Subsequently, 10 µL of fluorescence dye solution was dispensed into each well of the black, flat, clear‐bottomed, 384‐well plate (Corning Cat no. 3764), which already contained 10 µL of MB6 at various concentrations. The plate was incubated in the dark at 37 °C for 1 h. Afterward, the fluorescence intensity was monitored over a 6‐h period using a Cytation 5 multi‐mode plate reader (BioTek, USA), with excitation and emission wavelengths set at 490 and 520 nm, respectively. This assay was conducted in triplicate.

### All‐Atom Molecular Dynamics Simulations

Molecular dynamics (MD) simulations were performed to investigate the interactions between MB6 and a simulated bacterial plasma membrane based on the free and open‐source software GROMACS/2018.2.^[^ ^42^ ^]^ The topologies and parameters of MB6, compatible with the GROMOS54a7 force field,^[^ ^43^ ^]^ were computed using the Automated Topology Builder (ATB),^[^ ^44^ ^]^ which can be freely accessed on the website (https://atb.uq.edu.au/) using molid: 1223233. The plasma membrane of *S. aureus* was modeled as a mixed lipid bilayer comprising 88 DOPC and 40 DOPG lipids (≈7:3 ratio)^[^ ^8^, ^23^ ^]^ with Berger's lipid force field^[^ ^45^ ^]^ that is widely used in research.^[^ ^8^, ^21^, ^23^, ^46^ ^]^ The simulation setup has been detailed in our previous work.^[^ ^8^, ^21^, ^28^, ^29^ ^]^ Briefly, the SPC/E water model^[^ ^47^ ^]^ and the fast smooth particle‐mesh Ewald method^[^ ^48^ ^]^ were employed to calculate the long‐range electrostatic interactions. Forty sodium ions were included to maintain system neutrality. Our system had an initial size of 5.96×5.96×12.3 nm with periodic boundary conditions in all directions. The time step was 2 fs and NPT ensemble (1 atm, 300 K) was adopted. Post a 500 ns initial equilibration of solvated lipid systems, MB6 was introduced into the water phase above the membrane, followed by a further 100 ns re‐equilibration. On releasing MB6, the system was simulated for another 500 ns, recording the center of mass (COM) distance between MB6 and the lipid bilayer every 100 ps. Simulations were further conducted to derive the free energy profile associated with the penetration of MB6 into the membrane using steered MD,^[^ ^49^ ^]^ umbrella sampling, and the weighted histogram analysis method (WHAM).^[^ ^24^, ^50^ ^]^ Thermal energy, k_B_T, was used as the unit of energy. In umbrella sampling, each of the 42 windows was simulated for 25 ns (width: 0.1 nm), and the first 5 ns was discarded in the WHAM analysis.

### Phospholipid Binding Assay

The binding affinity of MB6 was evaluated based on changes in MIC values derived from the checkerboard microdilution assay as previously described.^[^ ^21b^ ^]^ Phospholipids including phosphatidylglycerol (PG), lysyl‐PG, and cardiolipin (Avanti Polar Lipids, Birmingham, AL, USA) were dissolved in methanol to prepare stock solutions at a concentration of 10 mg mL^−1^. In 96‐well plates, a range of concentrations was prepared for both MB6 and each phospholipid. For MB6, a twofold dilution series was prepared starting from 64 µg mL^−1^ along the *x*‐axis. Similarly, a twofold dilution series for the phospholipids was prepared starting from 64 µg mL^−1^ along the *y*‐axis. To each well containing 50 µL of the MB6 and phospholipid mixture, 50 µL of bacterial suspension at 1 × 10^6^ CFU mL^−1^ was added, yielding a final volume of 100 µL. Ciprofloxacin, known for its ability to penetrate phospholipid bilayers, was used as a negative control. Following an overnight incubation at 37 °C, OD_600_ was measured using a Cytation 5 multi‐mode reader (BioTek, USA). Bacterial growth was defined at OD600 ≥ 0.1. The experiments were independently replicated three times.

### Checkerboard Assay

The checkerboard method was used to determine the interactive effects of MB6 in combination with conventional antibiotics, as previously described.^[^ ^17^ ^]^ Briefly, an 8×8 matrix was created in a 96‐well microtiter plate by combining twofold serial dilutions of MB6 with twofold serial dilutions of each conventional antibiotics. Then, 50 µg mL^−1^ of 1×10^6^ CFU mL^−1^ *S. aureus* MW2 culture was added into each well of the assay plate including 50 µL. of antibiotic combinations. After incubating overnight incubation at 37 °C, OD_600_ was measured using a Cytation 5 multi‐mode reader (BioTek, USA). Bacterial growth was defined at OD600 ≥ 0.1. The fractional inhibitory concentration index (FICI) was calculated as follows: FICI = MIC of compound A in combination/MIC of compound A alone + MIC of compound B in combination/MIC of compound B alone. The interaction between two compounds was classified, as follows: synergy if FICI ≤ 0.5, no interaction if 0.5 < FICI ≤ 4, antagonism if FICI > 4.^[^ ^17^ ^]^

### Hemolysis Assay

Washed human red blood cells (RBCs) at a concentration of 25% were procured from Innovative Research (Novi, MI, USA). The RBCs were further diluted to a concentration of 4% using PBS. Subsequently, 100 µL of the diluted RBCs was added to 100 µL of twofold serial dilutions of MB6 in PBS, 0.2% DMSO (negative control), or 2% Triton X‐100 (positive control) in a 96‐well plate. The 96‐well plate was then incubated at 37 °C for 1 h, followed by centrifugation at 500 × *g* for 5 min. One hundred microliters of the supernatant was carefully transferred to a new 96‐well plate, and absorbance was measured at 540 nm. Hemolysis was quantified as a percentage using the following formula: (A_540_ of compound‐treated sample − A_540_ of 0.1% DMSO‐treated sample)/(A_540_ of 1% Triton X‐100‐treated sample − A_540_ of 0.1% DMSO‐treated sample) × 100. The experiments were independently repeated three times using different batches of human erythrocytes.

### Evaluation of ATP Leakage from Mammalian Cells

ATP leakage from the immortalized human embryonic kidney cell line, HEK‐293 cells, and the human hepatoma HepG2, in response to MB6 treatment, was assessed using the RealTime‐GloTM Extracellular ATP Assay (Promega, Madison, WI, USA). HEK‐293 cells were maintained in Dulbecco's Modified Eagle's Medium (DMEM, Gibco) with 15 mm HEPES, and HepG2 cells were cultured in DMEM/F‐12 (Gibco). Both media were supplemented with 10% fetal bovine serum and penicillin–streptomycin (100 units mL^−1^), and cells were incubated in a humidified 5% CO_2_ incubator at 37 °C. Once the cultures achieved 70–80% confluence, the cells were transferred to tissue culture‐treated 96‐well plates including 100 µL volume per well of the corresponding culture media. For the ATP leakage assessment, the cells were washed twice with PBS, followed by another wash with the corresponding culture media that were devoid of serum and antibiotics. Subsequently, the cells were exposed to varying concentrations of MB6 or 16‐BAC in the serum‐and antibiotics‐free culture media for 1 h. Post‐treatment, 33.4 µL of the 4× reagent mixture was added to each well, and the resultant luminescence was quantified using a Cytation 5 multimode plate reader (BioTek, USA). All experiments were performed in triplicate.

### Evaluation of Cytotoxicity

HepG2 and HEK‐293 cell lines were cultured in 96‐well tissue culture‐treated plates to achieve 70–80% confluence, using 100 µL of the corresponding culture media. Subsequently, the cells were treated with a range of concentrations of MB6 for 24 h in a humidified incubator set at 37 °C with 5% CO_2_. During the final hour of this 24‐h period, 10 µL of WST‐1 reagent (Sigma cat no. 5015944001) was added in each well. The reduction of WST‐1, indicative of cell viability, was measured at 450 nm using a Cytation 5 multimode plate reader (BioTek, USA). The results were expressed as a percentage of fluorescence relative to nontreated control wells. The assay was performed in triplicate. The median lethal concentrations (LC50) were determined using GraphPad Prism 10 software (GraphPad Software, Inc., La Jolla, CA, USA).

### Preparation of MRSA MW2 Persisters

MRSA MW2 persister cells were generated following the established arsenate treatment method.^[^ ^4^ ^]^ This approach employs arsenate to inhibit intracellular ATP synthesis, thereby facilitating the formation of *S. aureus* persisters.^[^ ^4^ ^]^ Initially, an overnight culture of *S. aureus* MW2 was diluted 1:10 000 into 25 mL TBS in a 250‐mL flask. The diluted culture was then incubated at 37 °C with shaking at 200 rpm until it reached exponential phase, indicated by an OD_600_ of 0.4. Sodium arsenate dibasic heptahydrate (Sigma cat no. A6756) was subsequently added to the culture to attain a final concentration of 5 mm, followed by an another 30‐min incubation at 37 °C with shaking at 200 rpm. After this, the arsenate‐treated MRSA cells were washed three times with PBS and resuspended with PBS to achieve an OD_600_ of 0.4. The successful isolation of MRSA persister cells was validated by determining cell viability after 4‐h treatment with 100× MIC of vancomycin, gentamicin, or ciprofloxacin.

### MRSA Biofilm Persister Killing Assay

*S. aureus* MW2 overnight culture was diluted 1:200 in TSB supplemented with 0.2% glucose and 3% NaCl. Each well of a 24‐well plate was furnished with a 13 mm mixed cellulose ester membrane (GSWP01300; EMD Millipore, USA), into which a 100 µL aliquot of the diluted culture was added. These samples were then statically incubated at 37 °C. After a 24‐h period, the biofilm‐forming membranes were washed three times with 1 mL PBS. Following the washes, 1 mL of PBS containing the indicated concentration of antibiotics was introduced to each well, and the plate was incubated at 37 °C for an additional 24 h. The membranes were then washed again three times with 1 mL PBS and relocated into 1.5‐mL microcentrifuge tubes filled with 1 mL PBS. After this, the samples underwent sonication in a Bronson ultrasonic bath for 10 min. These sonicated samples were serially diluted using PBS and spot‐plated on CaMH agar plates. The plates were incubated at 37 °C, post which the colonies were enumerated to estimate the number of surviving cells.

### Gentamicin‐Texas Red Uptake Assay

The uptake of the gentamicin conjugated with Texas Red was analyzed following an established method with minor modifications.^[^ ^51^ ^]^ Gentamicin‐Texas Red (GTTR) conjugates, purchased from AAT Bioquest (Cat no. 24 300, Pleasanton, CA, USA), were dissolved in DMSO to prepare a 5 mg mL^−1^ stock solution. MRSA persisters were subjected to a treatment involving 16 µg mL^−1^ GTTR in combination with indicated concentrations of compounds for 1 h at room temperature. The treated persister cells were then washed three times with PBS. 100 µL of these washed cells were transferred into a block, clear‐bottom 96‐well plate. Fluorescence intensity was assessed using a Cytation 5 multi‐mode reader (BioTek, USA) with an excitation wavelength of 589 nm and an emission wavelength of 615 nm. Experiments were conducted in biological triplicates.

## Conflict of Interest

The authors declare no conflict of interest.

## Author Contributions

H.Y.H., G.Z., E.M., F.M.A., H.G., and W.K. conceptualized the study; H.Y.H., G.Z., S.B., H.G. and W.K. contributed to methodology; H.Y.H., G.Z., S.B., H.G., and W.K. performed formal analysis; H.Y.H., G.Z., S.B., and W.K. performed investigations; J.K., E.M, H.G., and W.K. acquired resources; H.Y.H., G.Z., E.M., F.M.A., H.G., and W.K. wrote the manuscript; J.K., E.M, F.M.A., H.G., and W.K. acquired funding.; and W.K. supervised the study.

## Supporting information

Supporting Information

Supplemental Video 1

## Data Availability

The data that support the findings of this study are available from the corresponding author upon reasonable request.
