# Supplementary material for: A Methylazanediyl Bisacetamide Derivative Sensitizes Staphylococcus aureus Persisters to a Combination of Gentamicin And Daptomycin
Source: Adv Sci (Weinh). 2023 Dec 21;11(9):2306112. doi: 10.1002/advs.202306112 (PMC10916567; doi:10.1002/advs.202306112)
Supplement: Supplementary file 1 — Supporting Information [file ADVS-11-2306112-s001.pdf]

ADVANCED SCIENCE
Open Access

Supporting Information

for *Adv. Sci.*, DOI 10.1002/advs.202306112

A Methylazanediyl Bisacetamide Derivative Sensitizes *Staphylococcus aureus* Persisters to a Combination of Gentamicin And Daptomycin

*Hee Young Heo, Guijin Zou, Seongeun Baek, Jae-Seok Kim, Eleftherios Mylonakis, Frederick M. Ausubel, Huajian Gao and Wooseong Kim**

SUPPORTING INFORMATION

Table S1. Evaluation of cytotoxic potential of MB6

**Cell lines**	**HC50 or LC50 (μg/mL)**	**Selectivity/Therapeutic index (HC50 or LC50/MIC)**
Human erythrocytes	>256	>64
HEK-293	47	11.75
HepG2	40	10

HC50, median hemolytic concentration; LC50, median lethal concentration; MIC, minimum inhibitory concentration against *S. aureus* MW2

Table S2. Key Resources

REAGENT or RESOURCE	SOURCE	IDENTIFIER
Bacterial, nematode strains		
S. aureus MW2	ATCC	BAA-1707
S. aureus ATCC 33591	ATCC	ATCC 33591
S. aureus ATCC 43300	ATCC	ATCC 43300
S. aureus HL16278	Hallym University College of Medicine	BioSample Accession #: SAMN17301230
S. aureus HL17064	Hallym University College of Medicine	BioSample Accession #: SAMN17301231
S. aureus HL17078	Hallym University College of Medicine	BioSample Accession #: SAMN17301232
S. aureus HL18380	Hallym University College of Medicine	BioSample Accession #: SAMN17301233
S. aureus HL18807	Hallym University College of Medicine	BioSample Accession #: SAMN17301234
S. aureus HL18840	Hallym University College of Medicine	BioSample Accession #: SAMN17301235
S. aureus HL18883	Hallym University College of Medicine	BioSample Accession #: SAMN17301236
S. aureus HL18888	Hallym University College of Medicine	BioSample Accession #: SAMN17301237
S. aureus HL20835	Hallym University College of Medicine	BioSample Accession #: SAMN17301238
S. aureus HL21008	Hallym University College of Medicine	BioSample Accession #: SAMN17301239
S. aureus 0215	CDC & FDA Antibiotic Resistance Isolate Bank	AR Bank # 0215
S. aureus 0216	CDC & FDA Antibiotic Resistance Isolate Bank	AR Bank # 0216
S. aureus 0217	CDC & FDA Antibiotic Resistance Isolate Bank	AR Bank # 0217
S. aureus 0218	CDC & FDA Antibiotic Resistance Isolate Bank	AR Bank # 0218
S. aureus 0219	CDC & FDA Antibiotic Resistance Isolate Bank	AR Bank # 0219
S. aureus 0220	CDC & FDA Antibiotic Resistance Isolate Bank	AR Bank # 0220
S. aureus 0221	CDC & FDA Antibiotic Resistance Isolate Bank	AR Bank # 0221
S. aureus 0222	CDC & FDA Antibiotic Resistance Isolate Bank	AR Bank # 0222
S. aureus 0223	CDC & FDA Antibiotic Resistance Isolate Bank	AR Bank # 0223
S. aureus 0224	CDC & FDA Antibiotic Resistance Isolate Bank	AR Bank # 0224

S. aureus 0225	CDC & FDA Antibiotic Resistance Isolate Bank	AR Bank # 0225
S. aureus 0226	CDC & FDA Antibiotic Resistance Isolate Bank	AR Bank # 0226
S. aureus 0227	CDC & FDA Antibiotic Resistance Isolate Bank	AR Bank # 0227
S. aureus 0228	CDC & FDA Antibiotic Resistance Isolate Bank	AR Bank # 0228
S. aureus VRS1	(Kos *et al.*, 2012)	BioSample Accession #: SAMN02596782
E. faecium E007	(Garsin *et al.*, 2001)	
K. pneumoniae WGLW2	BEI Resources	HM-751
A. baumannii ATCC 17978	ATCC	ATCC 17978
P. aeruginosa PA14	(Rahme *et al.*, 1995)	BioSample Accession #: SAMN 33794324
E. aerogenes ATCC 13048	ATCC	ATCC 13048
C. elegans AU37	Caenorhabditis Genetics Center	AU37

Cells and cell lines		Cat#
Human red blood cell	Innovative Research	IWB3ALS40ML
HEK-293	Korean Cell Line Bank	21573
HepG2	Korean Cell Line Bank	88065

Chemicals and media		Cat#
Cation-adjust Mueller-Hinton Broth	BD	212322
Tryptic Soy Broth	BD	211825
Brain heart infusion broth	BD	237500
Agar	Sigma-Aldrich	A1296
HEPES (1M)	Gibco	15630080
FBS	Gibco	16000044
DMEM	Gibco	11965092
DMEM/F-12	Gibco	11320033
Dimethyl sulfoxide	Sigma-Aldrich	D2438
Methanol	Sigma-Aldrich	34860
Vancomycin	Sigma-Aldrich	V2002
Gentamicin	Sigma-Aldrich	G1264
Ciprofloxacin	Sigma-Aldrich	17850
Adarotene	Sigma-Aldrich	SML2061
Daptomycin	Tocris	3917
PQ401	Tocris	2768
Phosphatidylglycerol	Avanti Polar Lipids	841188P
Cardiolipin	Avanti Polar Lipids	841199P
Lysyl phosphatidylglycerol	Avanti Polar Lipids	840520P
MB1	Enamine	Z46395311
MB2	Enamine	Z46377505

MB3	Enamine	Z46395636
MB4	Enamine	Z46377518
MB5	Enamine	Z46384180
MB6	Enamine	Z46391767
SYTOX Green	Invitrogen	S7020
SYTOX Orange	Invitrogen	S11368
Triton X-100	Sigma-Aldrich	93443
WST-1	Sigma-Aldrich	5015944001

Critical Commercial Assays		Cat#
RealTime-Glo™ Extracellular ATP Assay	Promega	GA5012
Fluorometric Intracellular ROS kit	Sigma-Aldrich	MAK143

Garsin, D.A., Sifri, C.D., Mylonakis, E., Qin, X., Singh, K.V., Murray, B.E., *et al.* (2001) A simple model host for identifying Gram-positive virulence factors. *Proceedings of the National Academy of Sciences of the United States of America* 98: 10892–10897.

Kos, V.N., Desjardins, C.A., Griggs, A., Cerqueira, G., Tonder, A.V., Holden, M.T.G., *et al.* (2012) Comparative Genomics of Vancomycin-Resistant Staphylococcus aureus Strains and Their Positions within the Clade Most Commonly Associated with Methicillin-Resistant S. aureus Hospital-Acquired Infection in the United States. *mBio* 3: e00112-12-e00112-12.

Rahme, L.G., Stevens, E.J., Wolfort, S.F., Shao, J., Tompkins, R.G., and Ausubel, F.M. (1995) Common virulence factors for bacterial pathogenicity in plants and animals. *Science* 268: 1899–1902.

SUPPLEMENTARY FIGURES

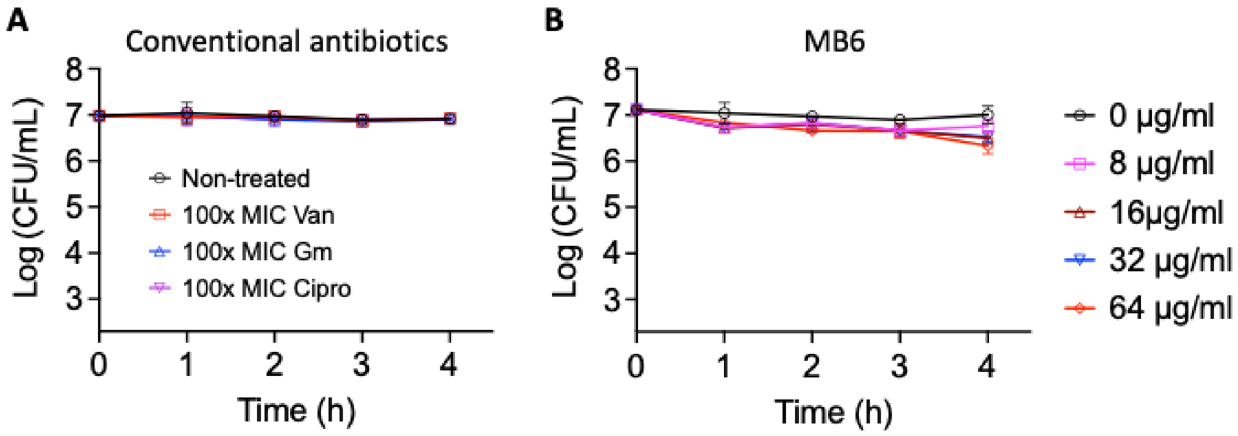

Figure S1. MB6 exhibits limited bactericidal activity against MRSA persisters**.** (A) MRSA MW2 persister cells were treated with 100× MIC conventional antibiotics or a range of concentration of MB6 for 4 h. Every hour viability was measured by serial dilution and plating on agar plates. Error bars represent means ± SD (n=3)

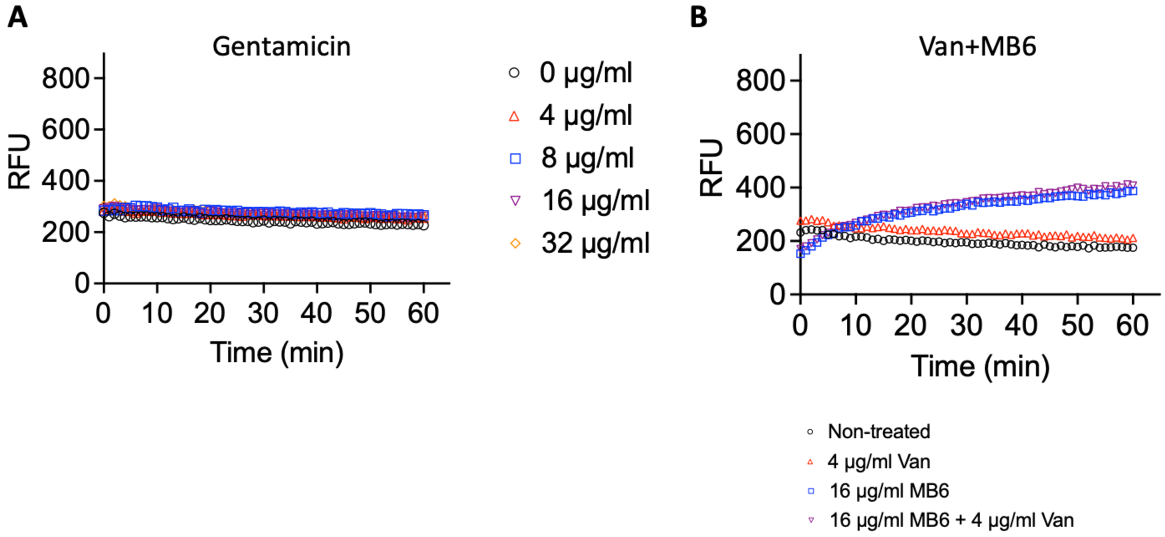

Figure S2. Gentamicin and vancomycin do not affect membrane permeability of MRSA persisters. Uptake of SYTOX Green (Ex = 485 nm, Em = 525 nm) by MRSA MW2 persister cells treated with varying concentration of gentamicin (Gm) (A) or the indicated concentrations of vancomycin (Van), MB6, or their combination (B). Results are shown as means (n = 3) Error bars are omitted for clarity.

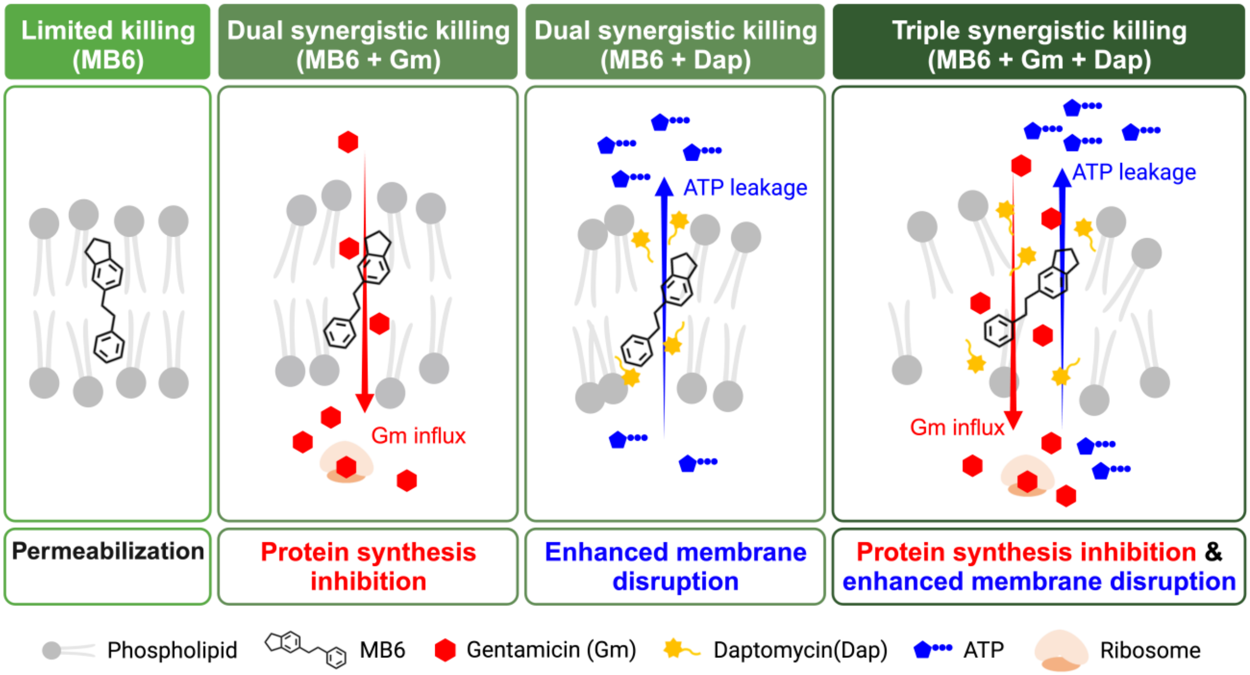

Figure S3. Schematic illustration of dual and triple synergistic interactions among MB6, gentamicin, and daptomycin.

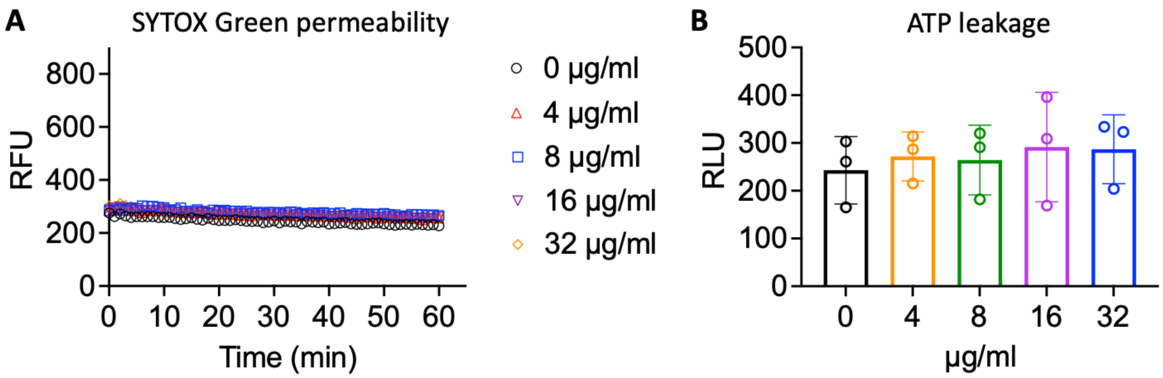

Figure S4. Daptomycin did not cause either membrane permeabilization or ATP leakage against MRSA persisters. (A) Uptake of SYTOX Green (Ex = 485 nm, Em = 525 nm) by MRSA MW2 persister cells treated with varying concentration of daptomycin. Results are shown as means (n = 3) Error bars are omitted for clarity. (B) ATP leakage from MRSA MW2 persister cells after treatment with varying concentrations of daptomycin. Individual data points are shown; error bars denote means ± SD (n = 3). Statistical differences were analyzed by one-way ANOVA and post hoc Tukey test.

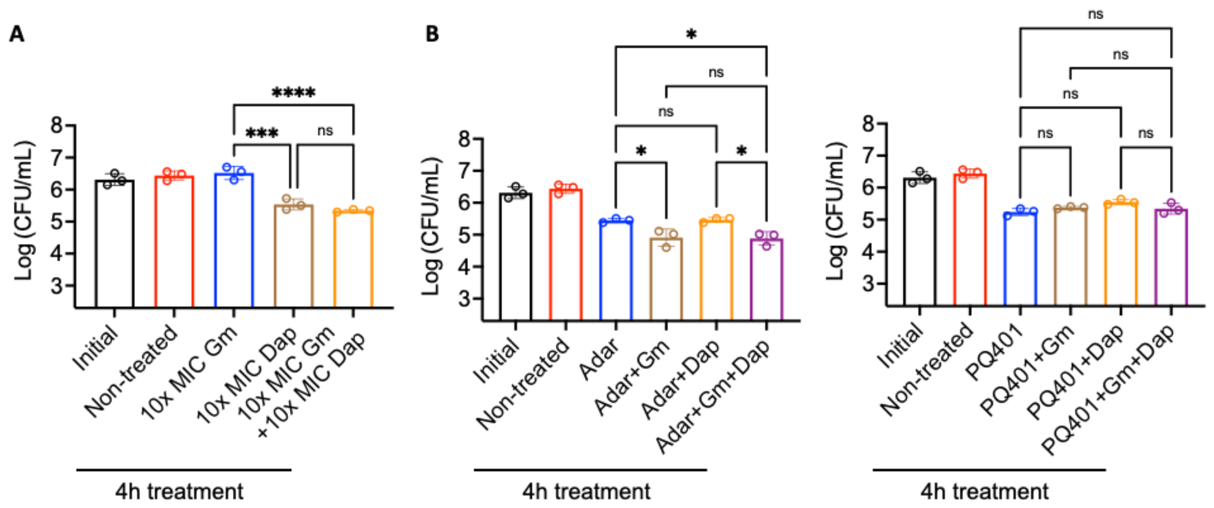

Figure S5. Adarotene and PQ401 do not potentiate the combined effect of gentamicin and daptomycin against MRSA persisters. (A) MRSA MW2 persister cells were exposed to 10× MIC (5 µg/mL) gentamicin (Gm), 10× MIC (10 µg/ml) daptomycin (Dap), or their combination for 4 hours. (B) The persister cells were treated with 2× MIC adarotene (Adar, (4 µg/mL) either alone, in combination with 2× MIC Gm (1µg/mL), with 2× MIC Dap (2 µg/mL), or both. Similarly, treatments included 2× MIC PQ401 (8µg/mL) either alone or in combination with 2× MIC Gm, 2× MIC Dap, or both Gm and Dap. All treatments were for 4 hours. Error bars represent means ± SD (n = 3). Each data point represents biologically independent samples. Statistical differences were analyzed using one-way ANOVA and post hoc Tukey test (* $P < 0.05$, *** $P < 0.001$, **** $P < 0.0001$).
